# One World, One Health: A growing need for an integrated global health approach

**DOI:** 10.4102/safp.v65i1.5693

**Published:** 2023-02-22

**Authors:** Ramprakash Kaswa, Klaus von Pressentin, Arun Nair, Shane Murphy

**Affiliations:** 1 Department of Family Medicine and Rural Health, Walter Sisulu University, Mthatha, Eastern Cape, South Africa; 2 Division of Family Medicine, Department of Family, Community and Emergency Care, Faculty of Health Sciences, University of Cape Town, Cape Town, South Africa; 3 Department of Family Medicine, Faculty of Health Sciences, University of the Free State, Kimberley, South Africa; 4 Robert Mangaliso Sobukwe Hospital, Northern Cape Department of Health, Kimberley, South Africa; 5 Mediclinic Pietermaritzburg, Mediclinic Southern Africa, South Africa

The increasing pressures on the human–animal–plant–environment interface contribute to the development of new and more complex diseases.^1^ In many cases, infectious disease outbreaks have strong links with environmental changes, such as the loss of biodiversity, the degradation of ecosystems and climate hazards,^2^ caused by anthropogenic activities. There is an urgent need to move from a global health perspective to an integrated ‘One Health’ approach. The coronavirus disease 2019 (COVID-19) pandemic has shown that the degradation of the environment is contributing to the increasing health risks faced by society. The emergence of the coronavirus in humans has highlighted the need for more effective and multi-disciplinary coordinated efforts.^1,3^ Furthermore, our collective responsibility is to prevent, predict, detect and respond to global health threats. Our current understanding of the various interactions between humans, animals, plants and the environment must be re-evaluated.

There is sufficient evidence supporting the notion that no single department or sector can effectively address the current challenges that affect One Health.^1,2,4^ One Health was developed to recognise that the health of humans, animals, plants and the ecosystem is interrelated.^5^ This framework aims to improve people, animals, plants and the environment’s health and well-being by integrating interlinked systems. It present a collaborative intersectoral approach that seeks to synergise systems while working towards achieving sustainable development goals (SDG).^4,6^

The concept of One Health was first established following the study of zoonoses.^5^ For decades, various international organisations have been working to address the multiple threats that can affect the human–environment interface. According to the One Health High-Level Expert Panel (OHHLEP):

One Health is an integrated, unifying approach that aims to sustainably balance and optimize the health of people, animals, and ecosystems. It recognizes the health of humans, domestic and wild animals, plants, and the wider environment (including ecosystems) are closely linked and interdependent.^7^

A primary health care system comprises three main components: comprehensive, integrated services focused on providing people with the best possible care and multi-sectoral policies that help address the various factors affecting their well-being. It also engages and empowers individuals and communities to improve their self-reliance in their environmental health context.^1^ One Health is a framework that aims to provide primary care providers with a comprehensive understanding of systems approaches applicable to various clinical settings. It expands the traditional concepts of inter-professional communication by incorporating ecosystem and animal health aspects.^2^ Getting the message across to primary care providers is not easy, especially because introducing One Health into their practice will require additional instruction in zoonotic infectious diseases, ecosystem and animal contact.^3^ A cross-sectoral communication and cooperation is essential for primary care providers to acquiring the One Health competencies to improve the community healthcare needs.^4^

Various global initiatives (for instance, the One Health Joint Plan of Action) have adopted the One Health approach to meet SDG and improve global health security.^4^ Through a participatory process, by creating a unified framework to integrate sectors and synergise capacities, One Health addresses health concerns (such as antimicrobial resistance and zoonotic diseases). The quadripartite organizations (World Health Organization [WHO], Food and Agriculture Organization of the United Nations [FAO], United Nations Environmental Programme [UNEP] and the World Organization for Animal Health [WOAH]) launched ‘One Health joint plan of action’ in October 2022 to strengthen and address health concerns at the human–animal–plant–environment interface. It also focuses on developing effective strategies to address the emerging and re-emerging threats of zoonotic diseases.

**FIGURE 1:**
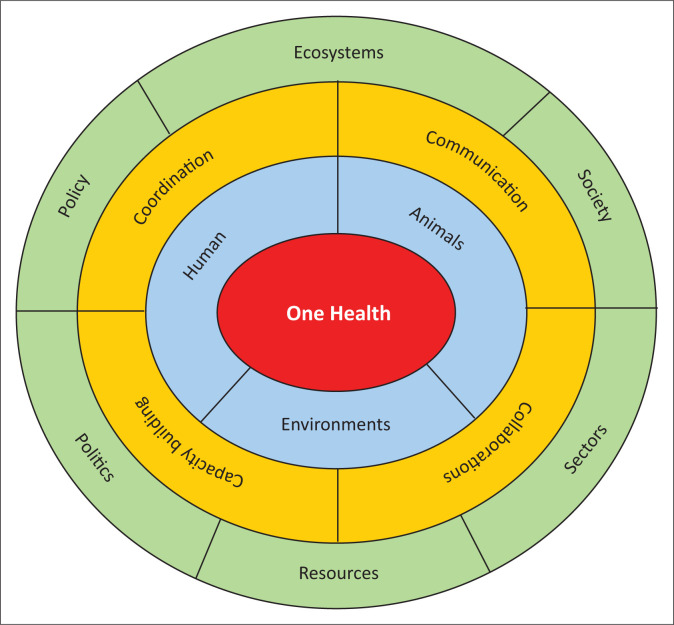
Theoretical framework of One Health approach.

Through its multiple components, One Health provides policymakers and other sectors of society with the necessary tools and resources to set national priorities and improve the quality of overall health.^8,9^ Figure 1 demonstrates the theoretical framework of the One Health approach.

Despite the efforts made by various sectors, the threat of infectious diseases outbreak remains. Different social and environmental shocks, such as natural disasters, the lack of proper environmental hygiene and the changes in the environment’s vector ecology, have increased the number of global disease outbreaks.^8^ These include the recent outbreaks of Ebola and monkeypox. In addition, the rising cost of healthcare is a burden that society’s most vulnerable individuals bear.^10^ The One Health approach aims to reduce these risks through a comprehensive approach to environmental, human and animal health. As the guiding principle of the new international agreement on pandemic preparedness, One Health is also expected to play a leading role in prevention and response efforts to outbreaks, epidemics and pandemics.^8,10^

Notwithstanding the various advantages of the One Health approach, its operationalisation and institutionalisation can still be challenging. This includes breaking down the silos that currently exist within multiple government agencies and non-governmental organisations. Factors that affect the coordination of the different sectors are the allocation of resources, training and education disparities and the environment’s various fields and disciplines.^4,7^ In addition, when implementing One Health control and prevention strategies, many sectors may not effectively integrate their efforts because of a lack of resources, incongruent budgeting procedures, and limited sharing of best practices. These factors can also affect the design and implementation of novel programmes.^4^

Unfortunately, implementing One Health in many African countries is hindered by the lack of proper coordination between the different sectors. Nevertheless, the African Centre for Disease Control and Prevention (CDC) believes that implementing One Health strategies is necessary to improve the efficiency and effectiveness of detecting, monitoring and controlling various infectious diseases and other health threats.^1,2^ Although the One Health approach is not a one-size-fits-all solution, it provides a valuable framework to address the multiple factors and programmes affecting a country’s health.^8^ Most African people rely on the primary health care system to meet their healthcare needs. It is within the system that primary care providers have to play a vital role in the management of human health by retaining a broader environmental perspective.^5^ Therefore, One Health should be integrated into community-orientated care so that all relevant sectors are working together efficiently to improve the quality of healthcare for the 21st century.
